# Experience in endoscopic resection of a mediastinal bronchogenic cyst penetrating the pleura into the thoracic cavity: first reported case

**DOI:** 10.1055/a-2552-0304

**Published:** 2025-05-09

**Authors:** Pingting Gao, Wei Yuan, Kaiqian Zhou, Danfeng Zhang, Quanlin Li, Pinghong Zhou

**Affiliations:** 1 92323 Endoscopy Center and Endoscopy Research Institute, Zhongshan Hospital Fudan University, Shanghai, China; 2 92323 Pathology, Zhongshan Hospital Fudan University, Shanghai, China

Bronchogenic cysts are rare and traditionally treated by surgical resection, which involves opening the pleura, causing an artificial pneumothorax and requiring chest drainage. Such methods, while effective, are invasive and associated with significant trauma, prolonged recovery, and high complication rates. With advancements in technology, endoscopic therapy for mediastinal tumors has emerged as a minimally invasive option 1 2 3 , and this case demonstrates a novel endoscopic approach that innovatively addresses the challenges of pleural penetration.

A young woman with a mediastinal mass (27 × 31 mm) underwent endoscopic resection ( Fig. 1 ). During the procedure, part of the cyst was found to be tightly adherent to the pleura, necessitating intentional pleural opening ( Fig. 2 **a–c** ). This caused the patient to experience temporary oxygen desaturation, which was controlled through anesthesia. After the cyst had been resected, the lung, diaphragm, and chest wall were visible through the defect ( Fig. 2 **d** ). The team restored pleural pressure using continuous suction combined with anesthetist-guided lung inflation before securely closing the endoscopic tunnel ( Fig. 2 **e, f** ). Importantly, no chest tube was used, with a gastric tube being inserted instead ( Video 1 ). The patient recovered rapidly, resuming a liquid diet on postoperative day 3 and being discharged on day 5 ( Fig. 3 **a** ). Follow-up confirmed a well-healed scar and no complications ( Fig. 3 **b** ).

**Fig. 1:**
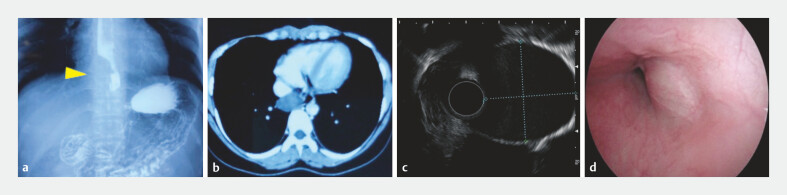
Appearance of the bronchogenic cyst on: **a** barium swallow, showing external compression in the lower esophagus with a localized filling defect; **b** chest computed tomography, showing a lesion at the lower end of the esophagus, which was thought possibly to be an esophageal cyst; **c, d** endoscopic ultrasonography, showing a submucosal tumor 28–36 cm from the incisors, measuring 27 × 31 mm, that was hypoechoic to anechoic, with flocculent echoes, indistinct boundary with the muscularis propria, and growing both intraluminally and extraluminally.

**Fig. 2:**
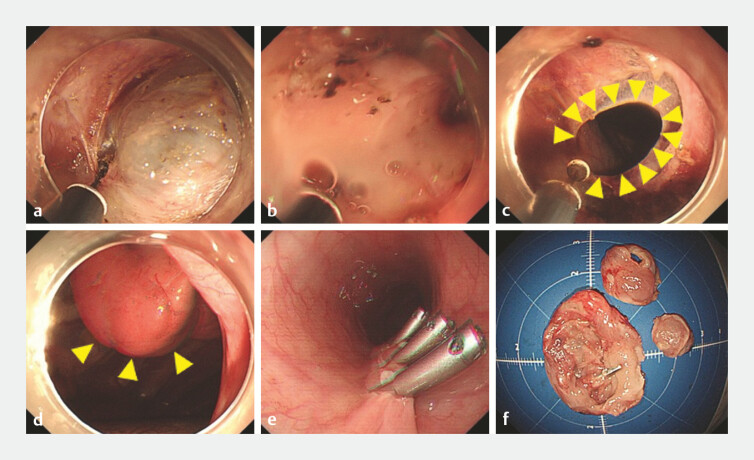
Images of the endoscopic resection showing: **a** incision of the esophageal muscle layer and exposure of the tumor; **b** dissection close to the tumor, which revealed a thin tumor wall, with a large amount of cyst fluid oozing from weak areas; **c** dense adhesion between the cyst wall and parietal pleura, necessitating unavoidable pleural opening for complete cyst wall removal; **d** the lung visible through the opening; **e** rapid closure of the tunnel opening with continuous suction; **f** the excised specimen, which was found on pathologic examination to be a bronchogenic cyst.

**Fig. 3:**
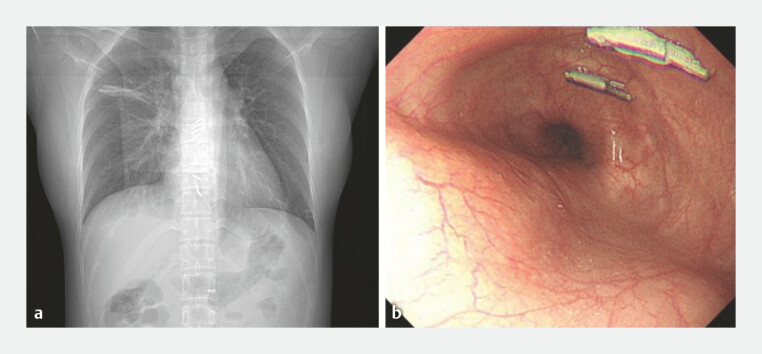
Follow-up examinations showing: **a** no atelectasis or pleural effusion on a chest radiograph on postoperative day 1; **b** a well-healed wound after 3 months, with the remaining metal clips being removed.

**Fig. 4:**
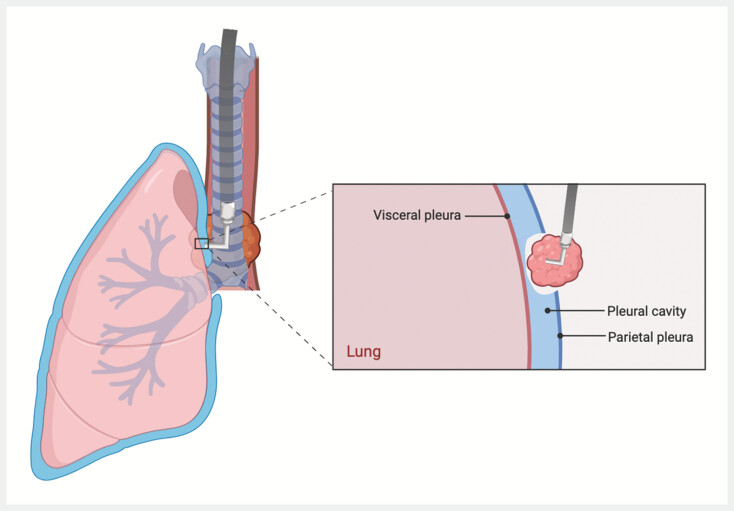
Illustration of the procedure.

A bronchogenic cyst that was adherent to the parietal pleura is successfully removed by penetrating the pleura from the mediastinum to thoracic cavity under endoscopic control, and managing wound closure by balancing the pressure, with postoperative drainage not required.Video 1

This groundbreaking approach introduces a minimally invasive technique for thoracic cavity procedures. It avoids postoperative complications, eliminates external wounds and foreign bodies, shortens hospital stays, and improves recovery. By actively managing pleural pressure during the operation and using the advantages of endoscopy, this method represents a significant step forward in thoracic endoscopic therapy, offering a viable alternative to traditional surgery ( Fig. 4 ).

Endoscopy_UCTN_Code_TTT_1AO_2AG_3AD

## Notes

Endoscopy E-Videos https://eref.thieme.de/e-videos
*E-Videos* is an open access online section of the journal *Endoscopy* , reporting on interesting cases and new techniques in gastroenterological endoscopy. All papers include a high-quality video and are published with a Creative Commons CC-BY license. Endoscopy E-Videos qualify for HINARI discounts and waivers and eligibility is automatically checked during the submission process. We grant 100% waivers to articles whose corresponding authors are based in Group A countries and 50% waivers to those who are based in Group B countries as classified by Research4Life (see: https://www.research4life.org/access/eligibility/ ). This section has its own submission website at https://mc.manuscriptcentral.com/e-videos .
